# Urinary and Faecal Incontinence: Point Prevalence and Predictors in a University Hospital

**DOI:** 10.3390/ijerph16020194

**Published:** 2019-01-11

**Authors:** Marie Condon, Edel Mannion, D. William Molloy, Rónán O’Caoimh

**Affiliations:** 1 Frailty Service, Department of Geriatric and Stroke Medicine, University Hospital Galway, Newcastle Rd, Galway City H91 YR71, Ireland; edel.mannion@hse.ie (E.M.); rocaoimh@hotmail.com (R.O.); 2 Physiotherapy Department, Cork University Hospital, Wilton, Cork City T12 DC4A, Ireland; 3 Centre for Gerontology and Rehabilitation, University College Cork, St Finbarrs Hospital, Douglas road, Cork City T12 XH60, Ireland; w.molloy@ucc.ie; 4 Clinical Research Facility Galway, National University of Ireland, Galway, Costello Rd, Galway City H91 V4AY, Ireland

**Keywords:** urinary, faecal, incontinence, prevalence, hospital, inpatient

## Abstract

Incontinence is common and associated with adverse outcomes. There are insufficient point prevalence data for incontinence in hospitals. We evaluated the prevalence of urinary (UI) and faecal incontinence (FI) and their predictors among inpatients in an acute university hospital on a single day. Continence status was recorded using the modified Barthel Index (BI). Baseline characteristics, Clinical Frailty Scale (CFS) and ward type were recorded. In all, 435 patients were assessed, median age 72 ± 23 years and 53% were male. The median CFS score was 5 ± 3. The point prevalence of UI was 26% versus 11% for FI. While UI and FI increased with age, to 35.2% and 21.1% respectively for those ≥85, age was not an independent predictor. Incontinence also increased with frailty; CFS scores were independently associated with both UI (*p* = 0.006) and FI (*p* = 0.03), though baseline continence status was the strongest predictor. Patients on orthopaedic wards had the highest prevalence of incontinence. Continence assessments were available for only 11 (2%) patients. UI and FI are common conditions affecting inpatients; point prevalence increases with age and frailty status. Despite this, few patients receive comprehensive continence assessments. More awareness of its high prevalence is required to ensure incontinence is adequately managed in hospitals.

## 1. Introduction 

Incontinence, both urinary and faecal, has negative effects on physical, emotional and social health [1,2,3,4]. It is common in community-dwellers, particularly among older adults [5]. Incontinence is associated with hospitalisation and subsequent adverse healthcare outcomes including increased direct and indirect costs to patients, carers and healthcare systems [6,7]. Hospitals in Europe, the United States and Australia have reported prevalence rates of urinary incontinence (UI) that range widely from 10 to 35% [8,9,10].

UI increases with age, affecting 30—40% of patients in geriatric wards in the United Kingdom [11] and Japan [12]. Faecal incontinence (FI) is also prevalent in the acute care setting with reported rates varying from 6.5% to 33% of inpatients [13,14,15]. Such variance in prevalence may be due to hospital setting, patient cohort and definitions of incontinence applied. Irrespective, rates are expected to increase in acute hospitals in keeping with international trends in population ageing. Despite this, there is insufficient prevalence data on incontinence in acute care settings including in Irish hospitals.

While common, incontinence is often stigmatised [16], even in hospitals. It is under-reported by patients and under-assessed and under-documented by healthcare providers [9,17,18,19]. Incontinence contributes to an accumulation of deficits, which negatively impact on patients’ physical function, frailty status, psychological well-being and social circumstances contributing to isolation and loneliness [1,2,3,4,10]. Adverse events associated with incontinence include longer length of hospital stay (LOS), greater odds of being discharged to a nursing home and increased mortality [4]. Furthermore, it is often incorrectly represented in hospital costings resulting in underfunding and underappreciation of its importance [9]. Given the significant impact of adverse outcomes and increased costs associated with incontinence on both the individual and healthcare systems [4,6], it is imperative to obtain a better understanding of the prevalence of incontinence in the acute care setting. 

The aim of this study was to determine the point prevalence and predictors of UI and FI in a large, acute university hospital.

## 2. Materials and Methods

A cross-sectional observational point prevalence study was conducted in a 742-bed university hospital in the west of Ireland. Data were collected over a 24 h period in August 2017. Consecutive sampling was used to record the continence status of all available English-speaking inpatients aged ≥18. Exclusion criteria consisted of patients deemed medically unstable according to nursing staff. Patients in the intensive care unit (ICU), coronary intensive care unit and high dependency unit (ICU step down) were automatically excluded due to the critical nature of their illness. Patients in the psychiatric and obstetric units were excluded as there are off-site from the general hospital and were not covered by the standard ethics approval obtained. Verbal informed consent was obtained from all individual participants included in the study and those who declined to participate were also excluded. Ethical approval was provided by the ethics committee of the Galway University Hospitals (Reference: C.A. 1806).

A brief patient interview and a review of patients’ medical and nursing notes was conducted using a specifically designed study assessment instrument to obtain patients’ current urinary and faecal continence status. The presence of a full continence assessment was sought. Nursing staff were asked to confirm the continence status where deemed necessary by the rater. Ward type and baseline demographics including age, sex, co-morbidities scored on the Combined Age-Charlson Co-morbidity Index (Charlson Index) [20], frailty scored on the Clinical Frailty Scale (CFS) [21] and function based on the modified Barthel Index (BI) [22] were also recorded. UI, the involuntary loss of urine, was recorded based on criteria on the BI whereby zero indicates incontinent or catheterized and unable to manage alone, one indicates occasional bladder accident and two indicates continence of urine [22]. FI, the involuntary loss of faecal liquid or loose stool, was recorded based on criteria included in the BI whereby zero indicates incontinent or needs to be given enemas, one indicates occasional accident and two indicates continence of faeces. In this study, UI and FI were defined by a current score of zero with those having occasional accidents regarded as continent. Baseline continence status was scored similarly. The BI is a tool designed to reflect patient’s abilities to perform activities of daily living (ADL) such that zero indicates complete dependency in ADL and a maximum score of twenty indicates complete independence in ADL [22]. The BI is widely used and has good inter-rater reliability (IRR) [23]. The Charlson Index is a validated scale of weighted comorbidities scored from one to six plus one point for every decade over 40 to measure burden of disease and predictor of mortality with good IRR [20]. The CFS is a validated frailty scale from one to nine whereby one indicates a robust individual and nine indicates terminally ill [21]. The scale has good IRR for assessing frailty and predicting inpatient mortality [24,25].

The study assessment instrument was piloted by the research team and amended based on feedback. Data were collected by 12 members of the geriatric medicine team. Data collectors were trained on the use of study assessment instrument. IRR was evaluated on a random sample of three patients reviewed by each data collector. A subsequent debrief session was conducted with raters to ensure standardisation of assessment and documentation.

Summary and descriptive statistics were calculated using IBM SPSS 25.0. Numerical data for age, BI and CFS were assessed for normality using the Shapiro Wilk test supported by Q-Q plots and found to be non-normally distributed. Median and interquartile ranges were therefore reported and compared using the Mann-Whitney U test. Distributions between categorical variables were compared with Chi-square tests. Logistic regression was used to examine the strength of relationship between variables.

## 3. Results

In all, 435 patients were included. Those excluded consisted of patients who were aged <18 (*n* = 2), too medically unstable (*n* = 1), non-English speaking (*n* = 1), patients off the ward (*n* = 9), where no collateral was available when required (*n* = 2) and those who refused consent to participate (*n* = 2). Continence status was incomplete for three patients. Six patients had incomplete ADL and frailty data but had data on incontinence and were included in the analysis. Patient demographics are summarised in Table 1. Patients had a median age of 72, ranging from 18 to 100 years and just over half, 53%, were male. The median CFS score was 5 interquartile range ± 3, indicating mild frailty [26]. Their median Charlson Index score was 5 ± 4. On the assessment day, the median length of stay was 9 ± 18 days. 

**Figure 1:**
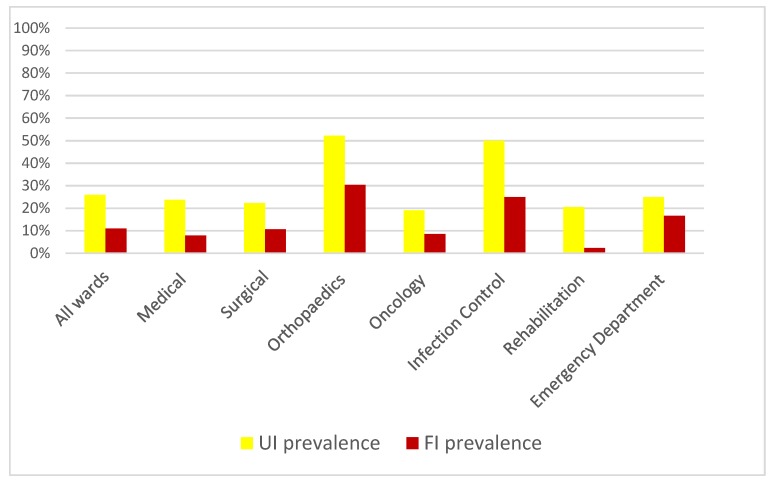
Point prevalence of urinary and faecal incontinence overall and according to ward type in an Irish university hospital. FI: Faecal Incontinence, UI: Urinary Incontinence.

The overall point prevalence of UI among current inpatients was 26% (111/435). Of these, 62 (55%) had urinary catheters in situ, yielding a point prevalence of UI without catheters of 13% (49/373). Occasional urinary accidents were reported by 12% of inpatients and 62% (268/435) reported continence of urine. Based on criteria agreed a priori, 74% (321/435) of patients were deemed continent of urine. The prevalence of FI was lower at 11% (46/435). Occasional faecal accidents were reported by 9% (37/435) while 80% (349/435) reported continence of faces ensuing 89% (386/435) of inpatients were continent of faeces. UI was more common in males (27%) than females (24%), albeit this was not statistically significant (*p* = 0.43). Similarly, FI was more common in female subjects but again not significantly different (*p* = 0.59). The presence of both UI and FI increased with age and frailty as presented in Table 2. Orthopaedic wards had the highest point prevalence of UI and FI (Table 3). Rehabilitation wards (off-site) had the lowest prevalence of FI, while oncology wards had the lowest prevalence of UI. The prevalence rates for each ward grouping are displayed on Figure 1 below. Only 2% (11/435) of inpatients had a formal continence assessment completed in their current nursing or medical records. Therefore, it was not possible to determine the prevalence of incontinence by classification or the reason for urinary catheter insertion. Likewise, although the main medical diagnosis and variables in the Charlson Index were recorded, genitourinary history was not recorded, meaning it was not possible to adjust for this or any other potentially contributing medical factors.

In the univariate analysis, age was statistically significantly associated with UI, with older patients more likely to be incontinent. The median age of those with incontinence was 76 versus 69 years for those without UI (*p* < 0.001). Those with FI were also more likely to be older (*p* < 0.001). Similarly, both UI (*p* < 0.001) and FI (*p* < 0.001) were significantly associated with higher CFS scores. The Charlson Index was also statistically higher for those with UI (median of 5 versus 6, *p* < 0.001) and FI than those without (median of 5 versus 6 respectively, *p* = 0.015). Those with a longer LOS at review were also more likely to have UI (7 versus 13 days, *p* = 0.005) and FI (8 versus 15 days, *p* = 0.012) than those without. Logistic regression examining the effects of age, gender, co-morbidity, length of stay and baseline continence showed that frailty status using the CFS (*p* = 0.0106) and UI status at baseline, based on the pre-admission BI score (*p* < 0.001), were independent predictors of UI on review. Baseline FI was the strongest predictor of current FI (*p* < 0.001); frailty status was also significant for FI (*p* = 0.03). The results of the logistic regression are presented in Table 4. 

## 4. Discussion 

This study describes the point prevalence of UI and FI among hospitalised adults. To our knowledge, this is the first to do so in an acute Irish university hospital and is also one of only a few papers to present point prevalence, particularly of FI, which is rarely reported. In this cohort, both UI and FI were prevalent conditions. The results suggest that while UI and FI are associated with age, co-morbidity and frailty status, that frailty, in this case measured using the Rockwood CFS, is an independent predictor even after adjusting for baseline continence. While the prevalence of UI was slightly higher in this study, the overall percentage was similar to self-reported percentages in other developed countries e.g., 22% of a convenience sample of 446 inpatients in Australia [9]. The prevalence of FI was equivalent at 11% here versus 10% in Australia [9]. However, the prevalence of both are lower than other studies providing estimates of between 30–40% [11,12]. There are several reasons for discrepancies. It must be noted that in this study patients with occasional episodes of UI were considered continent, which may lower the prevalence rates. In addition, a restricted definition of UI and FI was used, based on the BI. This said, the BI is a widely-validated and accepted measure of ADL, scored using self-reported and observations of the patient’s continence status. Further, patients with urinary catheters were deemed as incontinent based on the BI which may have increased the prevalence. One study found that 33% of patients categorised as incontinent were deemed so due to the presence of an indwelling catheter [27]. This is lower than that in this study (55%). Prevalence studies in Europe, which excluded the use of catheters revealed lower UI prevalence rates of between 10–20% [10], similar to the 13% found in this current study when catheters were excluded. 

The association between orthopaedic surgery and incontinence, whereby 21% of female inpatients post hip fracture developed UI, has been described previously [28]. While orthopaedic wards had the highest prevalence of UI in this study, the figure may be inflated due to the presence of post-operative urinary catheters. Furthermore, the study setting was the regional centre for trauma and orthopaedic services, which may influence the complexity of patients in the orthopaedic wards. Recently published multi-centre data on FI in acute care settings documented prevalence rates of 16% to 30% [15]. Our results were lower than this, though similar to those reported Austrian and Dutch hospitals of approximately 10% [10]. Such disparities may be due to the classification of those with ‘occasional accidents’ on the BI as not having FI and the exclusion of critical care wards in this current study. Patients in critical care are at high risk of developing FI [15]. Prevalence rates reported for FI may therefore be conservative while UI rates may be inflated. 

At a population level, female gender is associated with a higher prevalence of UI [5]. Contrary to this, prevalence rates of UI were higher among male inpatients in this current study while females demonstrated a higher prevalence of FI. Again, this likely relates to the higher proportion of men with urinary catheters, 65% (40/62). Research is conflicting in terms of sex and FI. Higher prevalence rates of FI have been noted among males in an acute care setting [27] while no significant gender difference was reported in a longitudinal study in an Austrian hospital [14]. Continence is a complex bodily function controlled through the interplay of a multitude of systems. Incontinence can be acute and transient or chronic and this is influenced by multiple factors in a hospital setting. It has been hypothesised that the disease pathogenesis of incontinence is different in men and women, which may influence the disparities in results in terms of gender [5]. Furthermore, the high prevalence of UI and urinary catheters among male patients may have been influenced by the high prevalence of urinary retention among older men [29]. As urinary catheters are used in the management of retention, figures need to be interpreted with caution as percentages may include not just those with UI but also in urinary retention.

Incontinence is considered to be an early marker for frailty [27] and our study is in keeping with results from a prospective cohort study which showed that UI is more common in frail patients even 12 months post discharge [2]. UI and frailty are also associated with higher mortality in the general population and in particular older adults [30]. UI increases the risk of mortality 3.4 times in the 12 months following hospitalisation [2]. The median score of five on the Charlson Index indicates a 34% 10-year survival rate [20]. The association between UI and mortality also increases with the severity of incontinence [29]. The type of UI and FI and the size (volume) of incontinence episodes were not obtained due to the lack of documentation in the patients’ medical and nursing notes. Continence is an integral component of a comprehensive geriatric assessment and provides vital prognostic information [30]. However, full continence assessments were only complete in 2% of inpatients in this study. Healthcare providers need to increase vigilance and routinely screen for incontinent episode before outcomes can be improved. 

This point prevalence study had a large sample size and low exclusion rates in an acute hospital. Rigorous efforts were made to ensure standardisation among data collectors to reduce any measurement error and information bias. Good compliance among data collections restricted missing data to only a small number of patients. Limitations of this study must however be acknowledged. Firstly, an observational point prevalence study was conducted. Causality between UI and the other variables measures cannot be ascertained. Hospitalisation is associated with new onset of UI [7] but the type of incontinence, its onset and any contributory factors were not explored. These may be confounding factors in the interpretation of the prevalence data. Further research is required to explore the role of such contributory factors and rates of incontinence in acute care settings. One must also be cognisant of the exclusion of a small number of acutely medically unwell patients and those in the ICU, coronary ICU and high dependency unit, some of whom would be at high risk of incontinence. Similarly, those in the psychiatric unit and obstetric unit would likely have had high rates. Prevalence rates may therefore be artificially altered (lowered). Finally, this data is from a single centre in one country, potentially limiting generalisability of results. This said, Irish hospitals and casemix are similar to that in the United Kingdom and other European countries.

## 5. Conclusions

Incontinence is a common condition among inpatients in a representative Irish hospital. The prevalence of UI and FI increased with age and frailty status. Frailty and baseline continence status were independent predictors of current incontinence. Considering the expected increase in older adults admitted to hospital and the detrimental effects of incontinence, routine identification of incontinence in patients, in particular older frail adults, is central to preventing adverse events. Further research is required to explore the relationship between factors such as medical condition, frailty and ward type and appropriate measures to address these. 

## Figures and Tables

**Table 1:** Baseline characteristics of inpatients included in the incontinence point prevalence study by current urinary and faecal continence status.

Variable	Total (*n* = 435 *)	UI (*n* = 111)	No UI (*n* = 324)	*p* = X (OR)	FI (*n* = 46)	No FI (*n* = 386)	*p* = X
**Age** (years)							
Median	72	76	69	*p* < 0.001	80	70.5	*p* < 0.001
(Q3 − Q1 = ±IQR)	(82 − 59 = ±23)	(83 − 67 = ±17)	(80 − 56 = ±24)		(89 − 71 = ±18)	(80 − 57 = ±23)	
**Gender**	54%	57%	52%	0.43	50%	54%	0.59
(% Male)	(233/435)	(63/111)	(169/324)	OR 1.2	(23/46)	(210/386)	OR 0.85
**Charlson Index**							
Median	5	6	5	*p* < 0.001	6	5	*p* = 0.015
(Q3 − Q1 = ±IQR)	(7 − 3 = ±4)	(8 − 4 = ±4)	(7 − 3 = ±4)		(8 − 5 = ±3)	(7 − 3 = ±4)	
**CFS** *							
Median	3	5	3	*p* < 0.001	6	3	*p* < 0.001
(Q3 − Q1 = ±IQR)	(5 − 2 = ±3)	(6 − 5 = ±1)	(4 − 2 = ±2)		(7 − 4 = ±3)	(4 − 2 = ±2)	
**Barthel Index** *							
Median	20	16	20	*p* < 0.001	13.5	20	*p* < 0.001
(Q − Q1 = ±IQR)	(20 − 17 = ±3)	(20 − 9 = ±11)	(20 − 19 = ±1)		(19 − 5 = ±14)	(20 − 18 = ±2)	
**Length of Stay**							
Median	9	13	7	*p* = 0.005	15	8	*p* = 0.012
(Q3 − Q1 = ±IQR)	(21 − 3 = ±18)	(42 − 4 = ±38)	(19 − 3 = ±16)		(60 − 4 = ±56)	(19 − 3 = ±16)	

CFS = Clinical Frailty Scale; FI = Faecal Incontinence; IQR = Interquartile Range; OR = Odds Ratio; UI = Urinary Incontinence. * Baseline values with missing data as described in the results section.

**Table 2:** Prevalence of Urinary Incontinence and Faecal Incontinence according to age and Clinical Frailty Scale score.

Variable *n* = 435 *		Total *n*	Urinary Incontinence	Prevalence (%)	Faecal Incontinence	Prevalence (%)
**Age**	Under 65	152	23	15.1%	7	4.6%
	65–74	104	27	26%	9	8.7%
	75–84	108	36	33.3%	15	13.9%
	Over 85	71	25	35.2%	15	21.1%
**Frailty**	Not Frail (CFS 1–3)	126	2	1.6%	0	0%
	Pre-Frail (CFS = 4)	79	6	7.6%	2	2.5%
	Mild to Moderate Frailty (CFS 5–6)	131	30	22.9%	7	5.3%
	Severe Frailty (CFS 7–9)	93	68	73.1%	35	37.6%

* Missing data as described in the results section.

**Table 3:** Prevalence of Urinary Incontinence and Faecal Incontinence according to ward type.

Ward Type *n* = 435 *	Total *n*	Urinary Incontinence *n*	Prevalence (%)	Faecal Incontinence *n*	Prevalence (%)
Medical	164	39	23.8%	13	7.9%
Surgical	121	27	22.3%	13	10.7%
Orthopaedic	23	12	52.2%	7	30.4%
Oncology	47	9	19.1%	4	8.5%
Infection Control	24	12	50%	6	25%
Rehabilitation	44	9	20.5%	1	2.3%
Emergency Department	12	3	25%	2	16.7%

* Missing data as described in the results section.

**Table 4:** Binary logistic regression model showing the association between variables and urinary and faecal incontinence.

Variable	B	Exp (B)	95% Confidence Interval	*p* = X
**Urinary Incontinence**				
Age	0.014	1.02	0.99–1.04	0.26
Gender	−0.540	0.58	0.33–1.03	0.06
Charlson Index	−0.006	0.99	0.87–1.13	0.92
Clinical Frailty Scale	0.276	1.32	1.08–1.61	0.006
Length of stay	0.004	1.00	0.99–1.01	0.17
Baseline urinary incontinence	−4.040	0.02	0.004–0.08	<0.001
**Faecal Incontinence**				
Age	0.028	1.03	0.99–1.07	0.14
Gender	0.007	1.01	0.47–2.18	0.99
Charlson Index	−0.056	0.95	0.79–1.14	0.55
Clinical Frailty Scale	0.285	1.33	1.02–1.73	0.03
Length of stay	0.001	1.00	0.99–1.00	0.59
Baseline faecal incontinence	4.374	79.3	9.36–672	<0.001
